# False-negative HHV-8 immunohistochemistry in Kaposi sarcoma: Clinicopathologic correlation leading to diagnosis of occult chronic lymphocytic leukemia

**DOI:** 10.1016/j.jdcr.2026.08.002

**Published:** 2026-08-17

**Authors:** Ghaith Al-Kuwari, Afraa Al Mohannadi, Wadha Al-Shafi, Hanof Ahmed, Salwa Al Maraghi, Wasim Akram, Sara Almeraghi, Yeslem Ekeibed, Mahir Abdulla Petkar, Nazeeha AlHayki, Badria Al Mahmoud, Sara Al-Khawaga

**Affiliations:** a Dermatology and Venereology Department, Hamad Medical Corporation, Doha, Qatar; b College of Medicine, Qatar University, Doha, Qatar; c Department of Geriatric Medicine, Rumailah Hospital, Hamad Medical Corporation, Doha, Qatar; d Department of Oncology, Hamad Medical Corporation, Doha, Qatar; e Department of Pathology, Hamad Medical Corporation, Doha, Qatar; f Clinical Dermatology, Weill Cornell Medicine-Qatar, Doha, Qatar; g College of Health and Life Sciences, Hamad Bin Khalifa University, Doha, Qatar

**Keywords:** chronic lymphocytic leukemia (CLL), HHV-8 immunohistochemistry, human herpesvirus 8 (HHV-8)-negative Kaposi sarcoma, Kaposi sarcoma, Kaposi sarcoma-associated herpesvirus (KSHV), small lymphocytic lymphoma (SLL)

## Introduction

Kaposi sarcoma (KS) is an uncommon vascular neoplasm that may affect the skin, oral cavity, lymph nodes, and internal organs. KS develops through the interaction of infection with Kaposi sarcoma-associated herpesvirus (KSHV), also known as human herpesvirus-8 (HHV-8), and immune dysregulation, both of which are essential for viral-driven tumorigenesis.^1^^,^^2^ Clinically, KS is characterized by violaceous, dark brown, or black macules, papules, and nodules, typically distributed on the lower extremities. KS has 4 clinical variants: classic, endemic, epidemic (HIV-associated), and iatrogenic.^1^ HHV-8 is linked to several other disorders, including multicentric Castleman disease (MCD), primary effusion lymphoma (PEL), and KSHV-associated inflammatory cytokine syndrome (KICS).^2^ KS associated with CLL/small lymphocytic lymphoma is uncommon compared with the classic and AIDS-related forms, with few cases reported in the literature (Table I). We report an HIV-negative elderly patient with KS and initial false-negative HHV-8 immunohistochemistry, later confirmed on repeat testing using a Ventana platform. Subsequent evaluation revealed occult CLL/small lymphocytic lymphoma, underscoring the need to consider underlying hematologic malignancy in patients without typical risk factors. This case underscores the importance of clinicopathologic correlation and repeat HHV-8 immunohistochemistry (IHC) when morphology strongly supports KS despite an initially negative stain.

**Table I:** Literature review of Kaposi sarcoma occurring in patients with hematologic malignancies

References	Age/sex	Diagnosis of lymphoma or leukemia/stage at KS diagnosis	Lymphoma/leukemia treatment prior to KS	Time interval (lymphoma/leukemia → KS)	HIV status	HHV-8 status	KS type	Proposed mechanism/authors’ interpretation
*Cutaneous KS*								
Hacioglu et al^3^	70/M	CLL, stage not reported	Rituximab + Fludarabine + Cyclophosphamide for 6 cycle	Not reported	Negative	Positive	Classic KS	Effect of immunodeficiency related to CLL
Kose et al^4^	64/F	CLL, stage not reported	Not reported	Not reported	Negative	Not reported	Classic KS	Latent HHV-8 infection can be reactivated in CLL patients and can trigger KS lesions.
Vučinić et al^5^	78/M	CLL,Rai stage 0	No prior treatment	7 years	Negative	Positive	Classic KS	Abnormalities in T-cell function
Tutaeva et al^6^	35/M	Nodular lymphocyte-predominant Hodgkin lymphoma (NLPHL), stage not reported	No prior treatment	Concurrent	Negative	Positive	Classic KS	Coinfection with multiple herpes viruses, can cause viral-driven immune dysregulation leading to KS and Hodgkin lymphoma development
Wang et al^7^	83/M	Extracavitary Primary Effusion Lymphoma (EC-PEL), stage not reported	No prior treatment	Not reported	Negative	Positive	Classic KS	Progression from KS to PEL can occur not only between distinct organs, but also within different regions of the same organ.
*Visceral KS*								
Ashman et al^8^	33/M	Intravascular lymphoma, stage not reported	Not reported	Concurrent	Positive	Positive	HIV-associated KS	KS and intravascular lymphoma tumorigenesis may involve HHV-8 infection, explaining their coexistence in HIV-associated immunosuppression
Meng et al^9^	51/M	Primary central nervous system lymphoma (PCNSL), stage not reported	No prior treatment	Concurrent	Positive	Not reported	HIV-associated KS	Severe HIV-induced immunosuppression/AIDS permitting viral oncogenesis causing KS and PCNSL.
*Lymph node KS*								
Licci et al^10^	59/M 58/M	DLBCL, stage not reported TCRBCL, stage not reported	Not reported Not reported	Concurrent Concurren0074	Positive Negative	Positive Positive	HIV-associated KS Classic KS	HHV8 DNA was detected in Kaposi sarcoma cells and EBV DNA in lymphomatous cells, suggesting independent oncogenic mechanisms
Zhang et al^11^	19 month (pediatric)/M	Post-transplant lymphoproliferative disorder (PTLD), stage not reported	Tacrolimus (FK506) + methylprednisolone	Concurrent	Negative	Positive	Iatrogenic KS	Immunodeficient status combined with EBV and HHV-8 coinfection can lead to PTLD and KS
Fernandes et al^12^	18/F	T-cell rich large B-cell lymphoma, stage not reported	Not reported	Concurrent	Negative	Positive	Endemic (African) KS	Concurrent HHV8 and EBV infection associated with development of KS and lymphoma by independent cellular mechanisms in the same lymph node
Lee et al^13^	81/F	Angioimmunoblastic T-cell lymphoma (AITL), Stage IV	No prior treatment	Concurrent	Negative	Positive	Classic KS	Immune dysregulation caused by AITL enables HHV-8 reactivation and the development of KS in the absence of HIV infection.
*AIDS associated KS*								
Bangar et al^14^	30/M	Primary effusion lymphoma, stage not reported	No prior treatment	Concurrent	Positive	Negative (serum PCR)	HIV-associated KS	AIDS-related immunosuppression
McKinnon et al^15^	48/F	Extra cavitary primary effusion lymphoma (EC-PEL), Ann Arbor stage IIIB	No prior treatment	Concurrent	Positive	Positive	HIV-associated KS	AIDS-related immunosuppression
Paksoy et al^16^	30/M	Diffuse large B-cell lymphoma (DLBCL), stage not reported	Not reported	Concurrent (same cervical lymph node)	Positive	Not reported	HIV- associated KS	AIDS-related cellular immune dysfunction may underlie the co-occurrence of Kaposi sarcoma with tuberculosis or diffuse large B-cell lymphoma in HIV-positive patients.

*AIDS*; Acquired immunodeficiency syndrome, *AITL*; angioimmunoblastic T-cell lymphoma, *CLL/SLL*; chronic lymphocytic leukemia/small lymphocytic lymphoma, *DNA*; deoxyribonucleic acid, *EBV*; epstein-barr virus, *HIV*; Human immunodeficiency virus, *EC-PEL*; extracavitary primary effusion lymphoma, *HHV-8*; human herpesvirus 8, *KS*; Kaposi sarcoma, *PCNSL*; primary central nervous system lymphoma, *PEL*; primary effusion lymphoma, *PTLD*; post-transplant lymphoproliferative disorder, *Rai*; scoring system for chronic lymphocytic leukemia.

## Case report

A 75-year-old woman with a history of stroke, dyslipidemia, prediabetes, osteoporosis with prior compression fracture, facial palsy, and mild cognitive impairment presented with an 8-month history of progressively enlarging skin lesions on the left foot, later involving the right. The lesions were asymptomatic. Examination showed multiple violaceous to brown patches and nodules on the left foot and violaceous macules and patches on the right. Dermoscopy demonstrated clustered violaceous nodules with a rainbow pattern, white intersecting lines, white clods, surface scale, and polychromatic color variation (Fig 1, *A* and *B*). Bilateral axillary lymphadenopathy was noted. Initial laboratory evaluation showed a complete blood count, including the absolute lymphocyte count, within the normal reference range, with no peripheral blood lymphocytosis or cytopenias, and a normal comprehensive metabolic panel. HIV and hepatitis B and C serologies were negative. Stool occult blood testing was negative.

**Fig 1:**
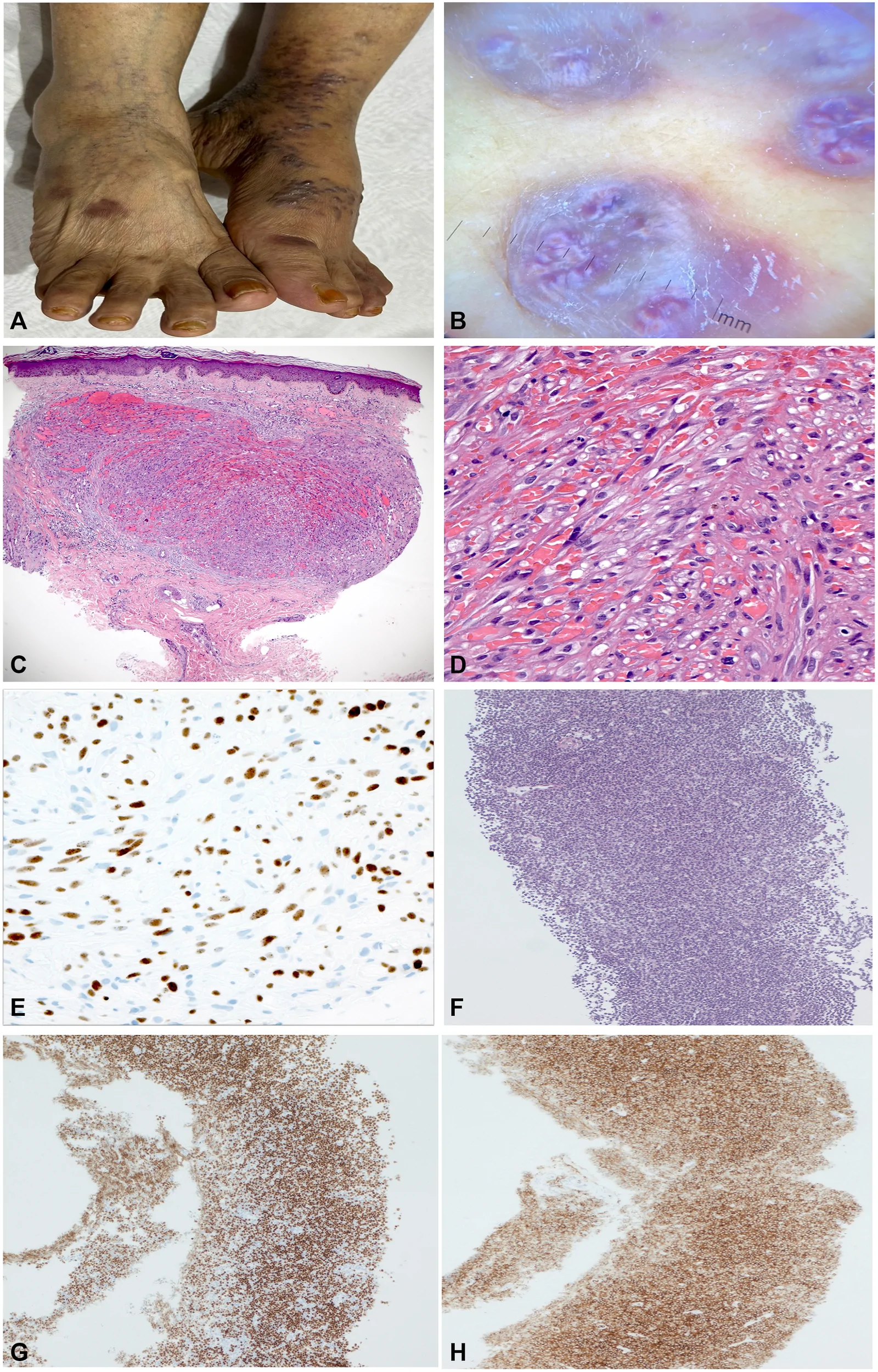
Clinical, dermoscopic, histopathologic, and immunohistochemical features of Kaposi sarcoma with associated chronic lymphocytic leukemia (CLL). **A,**Violaceous to brown patches and nodules on the dorsal aspects of both feet, more extensive on the left, with confluent violaceous plaques and nodules extending toward the plantar surface of the left foot and newer scattered brown-violaceous papules and macules on the right foot. **B,** Dermoscopic features of Kaposi sarcoma demonstrating clustered violaceous nodules with the characteristic rainbow pattern and overlying white intersecting lines. White lines and white clods, followed by surface scale and polychromatic color change, are noted. **C,** Skin punch biopsy showing histological features of Kaposi sarcoma, composed of characteristic slit-like vascular channels, spindle cell proliferation, lymphocytic inflammation and red cell extravasation (H and E × 4), **(D)** Higher power view of the lesion revealing prominent vascular spaces and spindled morphology of the tumor cells (H and E × 40), **(E)** HHV-8 immunohistochemistry showing the classical positive granular nuclear staining pattern, without any cytoplasmic staining. **F**, Lymph node biopsy showing a diffuse infiltrate of monomorphic small lymphoid cells (H and E × 10). **G,** PA**×**5 immunohistochemistry highlighting the neoplastic B-cell population. **H,** CD5 immunostain demonstrating diffuse positivity, including in the neoplastic B cells.

Three punch biopsies showed histopathologic findings consistent with Kaposi sarcoma (Fig 1, *C* and *D*), including characteristic slit-like vascular channels, spindle cell proliferation, lymphocytic infiltration, and extravasated red blood cells. However, initial HHV-8 immunostaining was negative (Cell Marque 13B10, Dako Omnis) on all 3 tissue blocks. Given the compelling clinicopathologic features, HHV-8 immunohistochemistry was repeated in-house on new sections from the same blocks, which remained negative, and subsequently at an external reference laboratory, where staining on the Ventana Autostainer platform demonstrated clear nuclear HHV-8 positivity, confirming the diagnosis of KS. HHV-8 immunohistochemistry was repeated in our laboratory using the Autostainer platform and was also positive. CD31 and ERG immunostains, performed on all 3 blocks, were strongly positive, supporting the vascular/endothelial lineage of the lesional cells.

Staging with PET/CT revealed mediastinal, axillary, and mesenteric lymphadenopathy, along with increased uptake in the left foot lesions without evidence of distant metastatic disease. Ultrasound-guided axillary lymph node biopsy demonstrated CD5 positive kappa-restricted monotypic B cells with immunophenotypic profile of CLL/small lymphocytic lymphoma, Binet stage B (Fig 1, *E-H*).

## Discussion

KS is an uncommon complication of chronic lymphocytic leukemia (CLL), occurring in both therapy-related and treatment-independent settings due to immune dysregulation. This case highlights a rare presentation in which KS had a false-negative HHV-8 immunohistochemistry and preceded a CLL diagnosis, suggesting that subclinical immune dysfunction may occur before overt disease.

### Diagnostic consideration: Platform-dependent false-negative HHV-8 immunohistochemistry in Kaposi sarcoma

• Platform-dependent variability in HHV-8 IHC can lead to false-negative results in KS, since sensitivity depends on detection chemistry, antigen retrieval, and signal amplification, not antibody clone alone.
• Methodologic differences between staining platforms, such as Dako Omnis and Ventana, may account for variability in antigen detection.^17^
• Prior reports describe IHC-negative KS cases confirmed by PCR, indicating that negative IHC does not exclude the diagnosis; therefore, in cases with characteristic histopathology, additional investigation is warranted.^18^^,^^19^

### Kaposi sarcoma in chronic lymphocytic leukemia: Therapy-related and intrinsic immune mechanisms

• KS has been reported following fludarabine monophosphate (FAMP) or chemotherapy, supporting a role for therapy-induced immunosuppression in HHV-8–mediated tumorigenesis.^3^^,^^20^
• Patients with CLL have an increased risk of secondary malignancies, with a reported 2 to 7-fold higher risk of KS among lymphoid malignancies.^21^^,^^22^
• KS can occur in untreated CLL, including in HIV-negative patients, suggesting that intrinsic immune dysfunction and impaired immune surveillance may contribute to disease development.^5^

### Diagnostic pitfall and clinical implications

• False-negative HHV-8 IHC may occur in Kaposi sarcoma; therefore, negative staining should not exclude the diagnosis when clinicopathologic features are characteristic, and additional diagnostic evaluation should be considered.^18^^,^^19^
• This case is notable for cutaneous KS preceding the diagnosis of CLL in the absence of HIV infection, immunosuppression, or laboratory abnormalities.
• The observed sequence supports the hypothesis that subclinical immune dysfunction associated with early CLL may permit HHV-8–driven tumorigenesis; KS may represent an early clinical clue to underlying immune dysregulation associated with occult CLL.
• Although causality cannot be definitively established, this case indicates that KS may rarely serve as the first clinical indicator of previously undiagnosed CLL, rather than a treatment-related complication.
• In older HIV-negative patients presenting with KS without typical risk factors, particularly in the presence of unexplained lymphadenopathy, clinicians should consider evaluation for an underlying lymphoproliferative disorder or occult malignancy.

## Conclusion

KS may rarely be the initial manifestation of undiagnosed CLL, even in HIV-negative patients without apparent immunosuppression. Negative HHV-8 IHC does not exclude KS in the presence of characteristic histopathology and should prompt further evaluation. Immune dysfunction inherent to CLL may precede therapy and increase susceptibility to HHV-8–associated neoplasms.

## Teaching points

• Negative HHV-8 immunohistochemistry does not exclude KS in the presence of characteristic histopathology and should prompt further evaluation.
• KS may rarely be the initial manifestation of previously undiagnosed CLL, including in HIV-negative patients without apparent immunosuppression.
• Immune dysfunction inherent to CLL may precede therapy and contribute to susceptibility to opportunistic or HHV-8–associated neoplasms.
• In older patients with KS without typical risk factors, particularly with unexplained lymphadenopathy, evaluation for an underlying lymphoproliferative disorder should be considered.

## Conflicts of interest

None disclosed.
